# Autotaxin and Endotoxin-Induced Acute Lung Injury

**DOI:** 10.1371/journal.pone.0133619

**Published:** 2015-07-21

**Authors:** Marios-Angelos Mouratis, Christiana Magkrioti, Nikos Oikonomou, Aggeliki Katsifa, Glenn D. Prestwich, Eleanna Kaffe, Vassilis Aidinis

**Affiliations:** 1 Division of Immunology, Biomedical Sciences Research Center “Alexander Fleming”, Athens, Greece; 2 Department of Medicinal Chemistry, University of Utah, Salt Lake City, Utah, United States of America; Chinese Academy of Sciences, CHINA

## Abstract

Acute Lung Injury (ALI) is a life-threatening, diffuse heterogeneous lung injury characterized by acute onset, pulmonary edema and respiratory failure. Lipopolysaccharide (LPS) is a common cause of both direct and indirect lung injury and when administered to a mouse induces a lung phenotype exhibiting some of the clinical characteristics of human ALI. Here, we report that LPS inhalation in mice results in increased bronchoalveolar lavage fluid (BALF) levels of Autotaxin (ATX, *Enpp2*), a lysophospholipase D largely responsible for the conversion of lysophosphatidylcholine (LPC) to lysophosphatidic acid (LPA) in biological fluids and chronically inflamed sites. In agreement, gradual increases were also detected in BALF LPA levels, following inflammation and pulmonary edema. However, genetic or pharmacologic targeting of ATX had minor effects in ALI severity, suggesting no major involvement of the ATX/LPA axis in acute inflammation. Moreover, systemic, chronic exposure to increased ATX/LPA levels was shown to predispose to and/or to promote acute inflammation and ALI unlike chronic inflammatory pathophysiological situations, further suggesting a differential involvement of the ATX/LPA axis in acute versus chronic pulmonary inflammation.

## Introduction

Acute lung injury (ALI), or mild acute respiratory distress syndrome (ARDS) [1], is a diffuse heterogeneous lung injury characterized by arterial hypoxemia, respiratory failure and low lung compliance, as well as non-cardiogenic pulmonary edema and widespread capillary leakage leading to alveolar flooding [2]. Different experimental animal models have been evolved and used to investigate the pathophysiological mechanisms of ALI, mostly based on reproducing known risk factors for the human condition, such as sepsis, acid aspiration and mechanical ventilation [3]. Among them, LPS inhalation in (C57Bl/6) mice is a well-established experimental model of ALI (LPS/ALI), characterized by acute neutrophil accumulation in lung tissue/BALF and pulmonary edema [4]. Lipopolysaccharide (LPS), a component of gram-negative bacteria cell walls and a potent TLR4 activator, is a common cause in both direct and indirect lung injury (i.e. pneumonia and sepsis respectively)[4].

Autotaxin (ATX, *Enpp2*) is a secreted glycoprotein, widely present in biological fluids, including broncheoalveolar lavage fluid (BALF) [5, 6]. ATX is a member of the ectonucleotide pyrophosphatase-phosphodiesterase family of ectoenzymes (E-NPP) that hydrolyze phosphodiesterase bonds of various nucleotides and derivatives [7]. However and unlike other E-NPP family members, the prevailing catalytic activity of ATX is the conversion of lysophosphatidylcholine (LPC) to lysophosphatidic acid (LPA)[8]. LPA is a phospholipid mediator [9, 10] that evokes growth-factor-like responses in almost all cell types, including cell growth, survival, differentiation and motility [11–13]. The large variety of LPA effector functions is attributed to at least six, G-protein coupled, LPA receptors (LPARs) with overlapping specificities and wide-spread distribution including the lung [14, 15].

A major role for the ATX/LPA axis has been suggested in chronic inflammation and cancer [16], while the numerous LPA effects in pulmonary cell types *in vitro* have implicated the axis in lung pathophysiology [17]. More importantly, genetic and pharmacologic studies *in vivo* [18–20] have indicated a decisive contribution of ATX/LPA in the development of pulmonary chronic inflammation and fibrosis [21–23]. Therefore, given the established role of the ATX/LPA axis in pulmonary chronic inflammation and fibrosis *in vivo*, as well as the LPA effects in pulmonary cell types *in vitro*, in this report we evaluated a possible role for the ATX/LPA axis in endotoxin-induced acute lung injury.

## Materials and Methods

### Mice

All mice were bred at the animal facilities of the Alexander Fleming Biomedical Sciences Research Center, under specific pathogen-free conditions. Mice were housed at 20–22°C, 55±5% humidity, and a 12-h light-dark cycle; water and food were given *ad libitum*. Mice were bred and maintained in their respective genetic backgrounds for more than 10 generations. All experimentation in mice for this project was approved by the Institutional Animal Ethical Committee (IAEC) of Biomedical Sciences Research Center “Alexander Fleming” (#373/375), as well as the Veterinary service and Fishery Department of the local governmental prefecture (#5508). The generation and genotyping instructions of *Enpp2* ^n/n^ conditional knockout mice [24], CC10-Cre [18] and LysM-Cre [25] have been described previously.

### Construction of the Tg*CC10hATX* transgenic mice

Vector pcDNA3.1/ZEO a1AT-hATX-BGHpA carrying the cDNA of human ATX preceded by the a1t1 promoter and followed by the Bovine Growth Hormone polyadenylation site (BGHpA) (a generous gift of G. Mills) was digested with HindIII/NaeI to isolate hATX-BGHpA. This was then ligated to pBS-CMV which had been cleaved with HindIII/EcoRV. The resulting vector pBS-CMV-hATX-BGHpA was digested with MfeI/HindIII to remove the CMV promoter and made blunt by filling the 5’ overhangs with T4 DNA polymerase. CC10 promoter was excised with a HindIII digestion from CC10-Cre-hGH and was also made blunt. Ligation followed, thus, forming the pBS-CC10-hATX-BGHpA construct. The resulting construct was verified with an MfeI/BssHII digestion, amplified in bacterial cultures and purified by 2xCsCl. BssHII (PauI) digestion was employed to separate the vector backbone from the transgene encoding fragment (transgenic device). The latter was then isolated by b-agarase extraction.

For the production of transgenic mice from the transgenic facility of BSRC Fleming, fertilized CBAxC57Bl/6 hybrid (F2) zygotes were injected with the transgenic device at a concentration of 5,38 ng/μl, diluted in Embryo max solution (mr-095-f, Chemicon International, CA, USA). In the same day, 233 zygotes were transferred to 11 surrogate mothers F1 CBAxC57Bl/6 to generate 54 offsprings.

The transgene was detected in tail DNA with PCR analysis (primers: forward 5´-ACT GCC CAT TGC CCA AAC AC-3´ and reverse 5’-TCT GAC ACG ACT GGA ACG AG-3’). From the 54 F0 mice 4 were identified as transgenic, which gave rise to the respective lines L13, L15, L16, L39.

### LPS-induced Acute Lung Injury Model

LPS was administered by inhalation, applying a previously described method with minor modifications [26, 27]. Briefly, bacterial LPS from *Pseudomonas aeruginosa* (serotype 10, Sigma, St. Louis, MO, USA) was dissolved in normal saline at a concentration of 2mg/ml. 5 ml of this solution was fully administered via a custom-made nebulizer at an oxygen flow-rate of 4lt/min for 25 minutes into a chamber containing 5–7 mice. For control mice, normal saline was administered as above. All measures were taken to minimize animal suffering; however and during the protocol no anaesthetics were used as no invasive or painful techniques were performed. After the induction of ALI, the condition of the animals was checked every two hours during the light period. No adverse effects were observed that would necessitate the use of analgesics and no animals died before the experimental points. Mice were sacrificed 24 hours after the induction of ALI for all experiments apart from the time course experiment where sacrifice was done at several time points between 6 and 48 hours after the induction. Sacrifice was performed in a CO_2_ chamber with gradual filling followed by exsanguination. In the pharmacologic study, GWJ-A-23 (dissolved in saline, 2% DMSO) was administered intraperitoneally at a dosage of 10mg/kg before exposure to LPS. The vehicle group was administered 2% DMSO in saline.

### Total Protein Content Determination

Total protein concentration in BALF samples was measured using the Bradford protein assay (Biorad, Hercules, CA, USA) according to the manufacturer’s instructions. OD readings of samples were converted to μg/ml using values obtained from a standard curve generated with serial dilutions of bovine serum albumin (2000–125 μg/ml).

### ATX activity assay

ATX activity was measured using the TOOS activity assay. ATX cleaves the LPC substrate to LPA and choline. The liberated choline is oxidised by choline oxidase to betaine and hydrogen peroxide. The latter, in the presence of HRP (horseradish peroxidase), reacts with TOOS (N-ethyl-N-(2-hydroxy-3-slfopropyl)-3-methylaniline) and 4-AAP (aminoantipyrene) to form a pink quinoeimine dye with a maximum absorbance at 555nm. Briefly, 1.25x LysoPLD buffer (0.12 M Tris-HCl pH = 9, 1.25 M NaCl, 6.25 mM CaCl_2_, 6.25 mM MgCl_2_, 6.25 μM CoCl_2_, 1.25 mM LPC) was incubated at 37°C for 30 minutes before adding 80μl/well in 20 μl BALF in a 96-well plate. The mix was incubated at 37°C for 4 hours. At the end of the incubation, a colour mix (5 mM MgCl_2_, 50 mM Tris-HCl pH = 8, 8 U/ml HRP, 0.5 mM 4-AAP, 0.3 mM TOOS, 2 U/ml choline oxidase) was prepared and 100 μl added to each well. Readings were taken every 5 minutes for 20 minutes. For each sample, the absorbance (A) was plotted against time and dA/min was calculated for the linear part of the plot. ATX activity was calculated according to the equation: Activity(u/ml) = [dA/dT(sample)—dA/dT(blank)] * V_t_/(32.8*V_s_*1/2), where Vt = total volume of reaction (mL), Vs = volume of sample (mL), 32,8 = the milimolar extinction coefficient of quinoneimine dye (cm^2^/μmol) and 1/2 = the mols of quinoneimine dye produced by 1 mol of H_2_O_2_.

### Immunohistochemistry

Immunostaining was performed with peroxidase labelling techniques. Tissue sections were deparaffinized and endogenous peroxidase activity was blocked by incubation in 1% peroxide. The sections were preincubated with 2% Normal Goat Serum (NGS) in PBS-Tween (PBST) for 30 minutes, followed by incubation overnight at 4°C with the primary antibody against ATX (Cayman Chemical Company, Michigan, USA). Sections were then washed in PBST and incubated for 30 minutes with horseradish peroxidase (HRP)–conjugated anti-rabbit IgG (1:1000 dilution in PBS-T). The sections were further washed with PBST. Finally, colour was developed by immersing the sections in a solution of 0.05% 3, 3’-diaminobenzidine (DAB; Sigma) and 0.01% hydrogen peroxide in PBS. The sections were counterstained with hematoxylin. The specificity of a-ATX antibodies has been analysed in detail previously [28].

### RNA Extraction and Real-time RT-PCR Analysis

RNA was extracted from the left lung lobe using the peqGOLD TriFast Reagent and treated with DNAse (RQ1 RNAse-free DNAse, Promega, Wis, USA) prior to RT-PCR according to manufacturer’s instructions. Reverse transcription was performed for cDNA synthesis using the peqGOLD MMLV H plus reverse transcriptase. All reagents were purchased from PEQLAB Biotechnologie GMBH, Germany. Real-time PCR was performed on a BioRad CFX96 TouchReal-Time PCR Detection System (Bio-Rad Laboratories Ltd, CA, USA). Values were normalized to the expression of b-2 microglobulin (B2m).

### HPLC-MS/MS measurements

LPA (C14:0, C16:0, C18:0, C18:1 and C20:4) and LPC species (C14:0, C16:0, C18:0, C18:1, C20:4, C22:6 and C24:0) were measured in plasma by means of HPLC-ESI/MS/MS using an RSLCnano system (Ultimate 3000 Series, Dionex Corporation, USA) coupled with an LTQ Orbitrap XL mass spectrometer (Thermo Scientific, Waltham, MA, USA). Lipid extraction from BALF was performed as previously described with minor modifications [29]. Briefly, BALF samples (300 μL) were mixed with 700 μL PBS prior to extraction and spiked with the internal standard mix (17:0 LPA/LPC). Neutral extraction was performed twice with 2 mL ice-cold CHCl3/CH3OH (2/1, v/v) followed by 1 mL PBS saturated ice-cold CHCl3/CH3OH (2/1, v/v). Each extraction step was followed by a 60 sec vortex and a 1 min centrifugation step at 4°C at 3,000 rpm. The lower organic phases from both extraction steps were pooled and kept for LPC measurements. The remaining aqueous phase was chilled in ice for 10 min, acidified with HCl 6N το pH 3.0 and undergone further 2-step extraction with ice-cold CHCl3/CH3OH (2/1, v/v) as above. The lower organic phases were pooled and kept for LPA measurements. The neutrally extracted organic phase and the neutralized acidified lower organic phase were evaporated to dryness. Finally, the dry residues were resuspended in 0.15 mL of isopropanol for HPLC-ESI/MS/MS analysis. Recovery of LPA and LPC species ranged between 55–85% and 80–100%, respectively. The HPLC-MS/MS was performed as previously described [29].

### Statistical analysis

Statistical significance was assessed in pair-wise comparisons with control values using a paired Student’s *t*-test, or a Mann-Whitney test in cases of not normal distributions, using SigmaPlot 11.0 (Systat software Inc., IL, USA), and presented as means (± S.E). In all figures, * and ** denote p-values <0.05 and <0.001 respectively. All experiments presented are representative of two repetitions; cumulative normalized-to-control values produce identical results and conclusions.

## Results and Discussion

ATX was previously suggested as a candidate gene involved in the control of pulmonary functions, development and remodelling [30], while the lung has been suggested to be among the tissues expressing moderately high ATX mRNA levels in healthy conditions [17]. In pathophysiological conditions, increased ATX/LPA levels have been detected in fibrotic lungs, both in human patients and animal models [18, 20]. Accordingly, genetic deletion of ATX, LPAR1 or LPAR2 [18, 20, 31], as well as pharmacologic inhibition of ATX or LPAR1[18, 19], attenuated the development of the bleomycin (BLM)-induced modelled disease. Therefore, a major role of ATX and LPA in pulmonary chronic inflammation and fibrosis was established, attributed to LPA-induced vascular leak and fibroblast recruitment [21–23]. Moreover, a number of LPA effects in pulmonary cells *in vitro* are consistent with a pro-inflammatory and pro-fibrotic role of ATX/LPA, although a number of reports also suggest anti-inflammatory effects of LPA [17]. Therefore, and given the role of ATX/LPA in chronic pulmonary inflammation and the LPA effects in pulmonary cell types, we reasoned a possible role of ATX/LPA in acute inflammation and lung injury.

### Increased BALF ATX/LPA levels upon LPS-induced ALI

**Fig 1:**
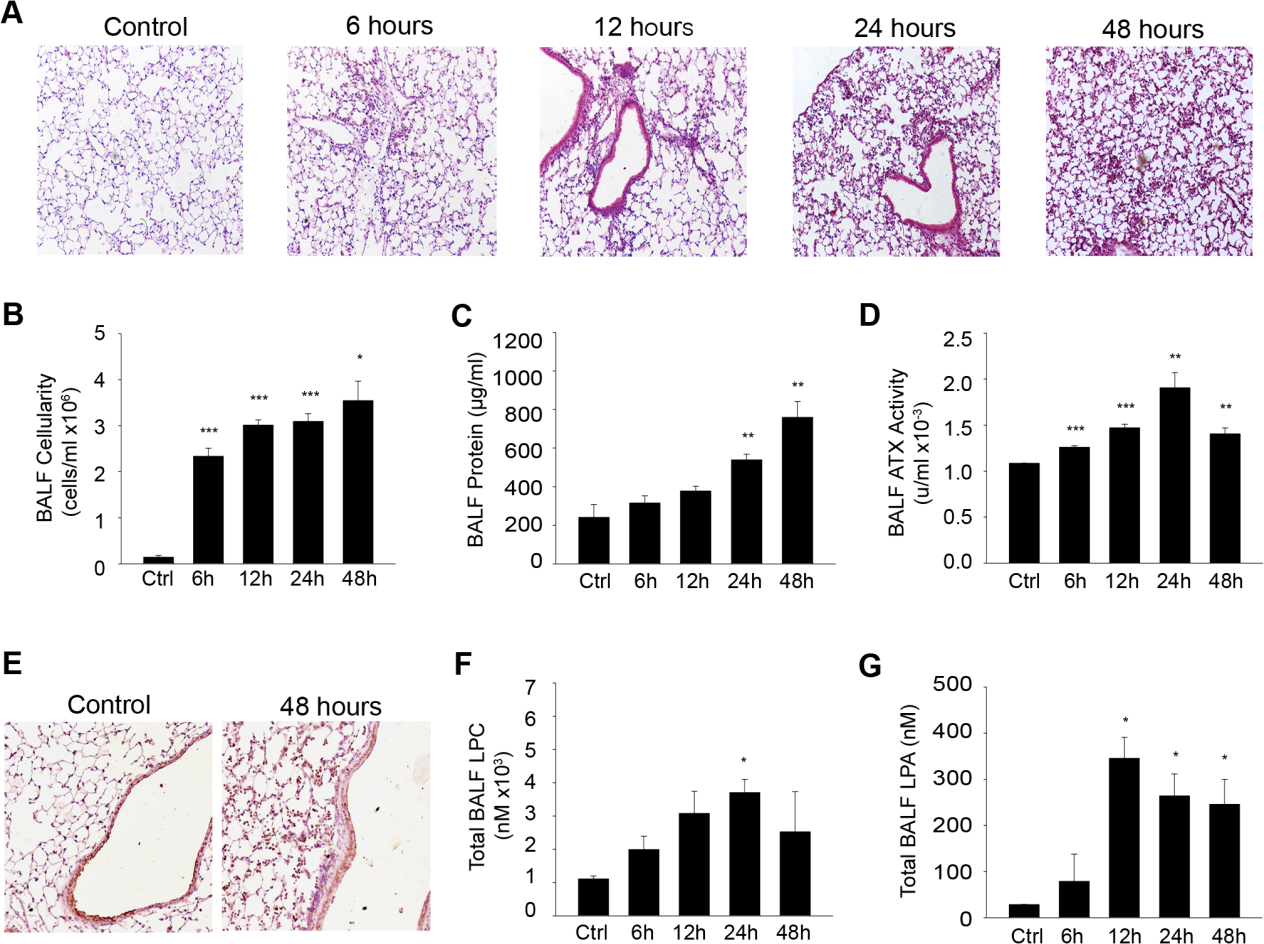
Increased BALF ATX/LPA levels upon LPS-induced ALI. Mice were administered aerosolized LPS and were sacrificed 6, 12, 24 and 48 hours later (n = 3–5; a representative experiment out of two is shown). A. Hematoxylin & Eosin staining of lung tissue sections following LPS exposure for the indicated times resulted in alveolar wall thickening and leukocyte infiltration into the lung interstitium and alveolar space. B. Increased BALF cellularity upon LPS/ALI. C. Pulmonary microvascular leakage and edema induced by LPS was reflected in BALF protein content. D. Increased ATX activity in LPS/ALI BALFs, as measured with the TOOS assay. E. IHC for ATX in lung tissue showing constitutive expression from the bronchial epithelium, as well as a weak diffuse staining pattern in the lung parenchyma. F-G. BALF total LPC/LPA levels respectively upon LPS/ALI, as measured with HPLC-MS/MS.

To examine a possible involvement of ATX in the pathogenetic mechanisms underlying ALI, we first monitored its levels upon the time course of the modelled, LPS-induced, disease development. Noteworthy, and since no animal model fully represent all the clinical characteristics of human ALI, a recent American Thoracic Society workshop suggested that the main features of experimental ALI should include at least three out of the following four features: histological evidence of tissue injury (such as the accumulation of neutrophils in the alveolar or the interstitial space), alteration of the alveolar capillary barrier (such as the increase in total protein concentration of the bronchoalveolar lavage fluid; BALF), an inflammatory response (such as an increase in the absolute number of neutrophils in the BALF) and evidence of physiological dysfunction [32]. Accordingly, aerosolized LPS (*Pseudomonas aeruginosa*) was administered by inhalation to groups of littermate mice, which were then sacrificed 6, 12, 24 and 48 hours post-administration (Fig 1). Histological analysis of isolated lungs indicated that LPS inhalation resulted in alveolar wall thickening and leukocyte infiltration into the lung interstitium and alveolar space (Fig 1A), as previously reported [26, 27]. Inflammatory cells (93% neutrophils; [26]) were evident in BALFs already at the 6hr time-point and continued to increase at 48h (Fig 1A and 1B). Pulmonary microvascular leakage and edema induced by LPS was reflected in the gradual increase of BALF total protein content (Fig 1C).

Interestingly, ATX activity in BALFs, as quantified with the TOOS assay on natural LPC substrates, showed a gradual increase as time progressed (Fig 1D), following total protein levels (Fig 1C) most likely reflecting a relaxation of the endothelial barrier and thus suggesting increased recruitment from the circulation. Similar findings have been reported in earlier studies, upon intratracheal administration of LPS (5mg/Kg) in Sv/129 mice [33]. ATX immunohistochemistry (IHC) in lung tissue sections showed high constitutive expression from the bronchial epithelium, as well as a weak diffuse staining pattern in the lung parenchyma upon LPS/ALI (Fig 1E).

As the main known function of ATX is the hydrolysis of LPC to LPA, the corresponding BAL fluids were analyzed with HPLC-MS/MS to identify perturbations in lysophospholipid levels upon LPS-induced ALI. LPC, the substrate of ATX and precursor of LPA, peaked at 24 hours (Fig 1F), as previously reported for the lung surfactant of guinea pigs upon LPS-induced ALI [34]. Total BALF LPA levels were also found increased (Fig 1G), as previously reported [35], correlating with and confirming BALF ATX activity levels.

### Bronchial epithelium-specific ATX expression has a minor contribution to ALI pathogenesis

ATX expression is localized mainly in the bronchial epithelium, both in healthy and inflammatory conditions (Fig 1E)[17, 18, 36], suggesting this cell type as the main pulmonary source of ATX. To confirm both its cell-specific expression, as well as to examine its possible contribution to ALI pathogenesis, ATX was conditionally deleted from the bronchial epithelium by crossing the conditional knock out mouse for ATX (*Enpp2* ^*n/n*^)[24] with the Tg*CC10-Cre* transgenic mouse strain that expresses the Cre recombinase under the control of the mouse CC10Kd (*Scgb1a1*) promoter [18]. As previously reported, CC10-Cre drives conditional ATX recombination in *CC10Enpp2* ^-/-^ mice exclusively in bronchial epithelial cells (with an efficiency of 70–80%), while the transgenic Cre driver mouse strain itself exhibits no apparent pulmonary phenotype even under inflammatory conditions [18].

**Fig 2:**
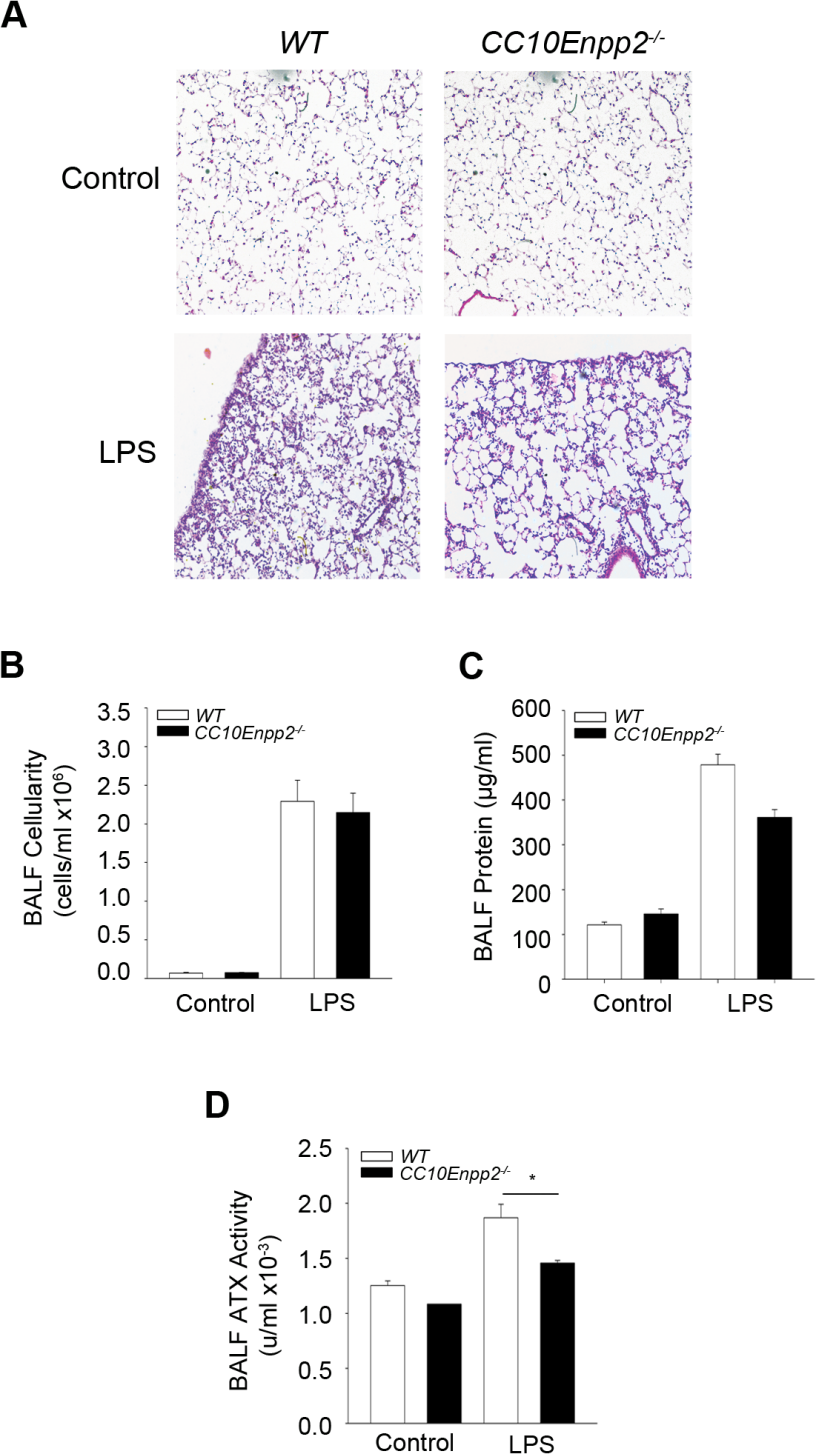
Genetic deletion of ATX from the bronchial epithelium has minor effects in ALI development. Aerosolized LPS was administered in mice where ATX was genetically deleted specifically from bronchial epithelial cells (CC10Enpp2^-/-^), as well as wild type littermates. Mice were sacrificed 24 hours later (n = 3–8; a representative experiment out of two is shown). A-C. Histological analysis (A) and BALF measurements (B & C) indicated no significant changes in the lungs of mice lacking ATX expression in the bronchial epithelium in comparison to their wild type littermates. D. Conditional deletion of ATX from the cells of the bronchial epithelium led to decreased enzyme activity of ATX in the BALF.

LPS was administered to *CC10Enpp2* ^-/-^ mice, as well as to wild type littermates, and disease severity was assessed 24 hours later. Deletion of ATX from bronchial epithelial cells had minor effects in attenuating disease development (a clear and reproducible, but not statistically significant, negative trend), such as tissue damage (Fig 2A), neutrophilic inflammation (Fig 2B) and pulmonary edema (Fig 2C), despite decreased BALF ATX levels (Fig 2D).

**Fig 3:**
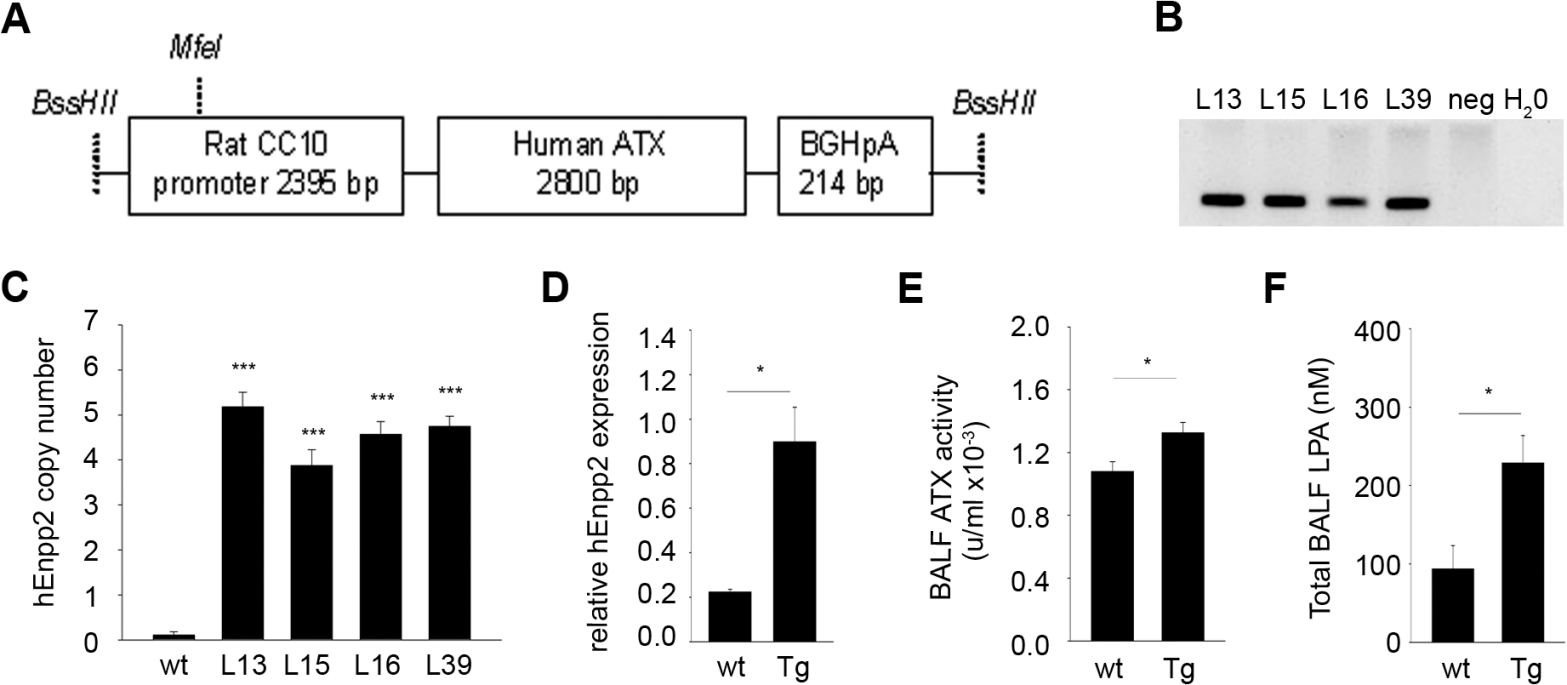
Generation of Tg*CC10Enpp2* mice. A. Schematic representation of the construct used for the generation of the transgenic mice. B. Genotyping PCR of the 4 offsprings that carried the transgene, out of the 54 that were generated after the injections of the trangene-microinjected zygotes in surrogate mothers. C. All four transgenic lines contained equal copy numbers, as identified with Real-Time PCR. D. Real-Time RT-PCR confirmed the expression of the transgene (L39 is shown). E. Total ATX activity levels in the BALFs of Tg*CC10Enpp2* mice (L39) were found moderately upregulated with the TOOS assay. F. In the same mice, BALF LPA was also found elevated, as measured with HPLC-MS/MS. (C-F n = 3–8).

In order to further examine a role of bronchial epithelial cell-derived ATX expression, a new transgenic mouse strain was constructed, expressing human ATX driven by the CC10 promoter (Tg*CC10hEnpp2*; Fig 3A) which directs expression exclusively in bronchial epithelial cells [18]. All 4 transgenic lines obtained (L13, L15, L16, L39; Fig 3B), contained 4–5 transgene copies (Fig 3C), and expressed the transgenic (hATX) mRNA in the lung tissue (Fig 3D; L39 is shown). Total ATX activity in BALFs of the transgenic mouse line was found moderately upregulated (Fig 3E), resulting in similar increases in BALF LPA levels (Fig 3F).

**Fig 4:**
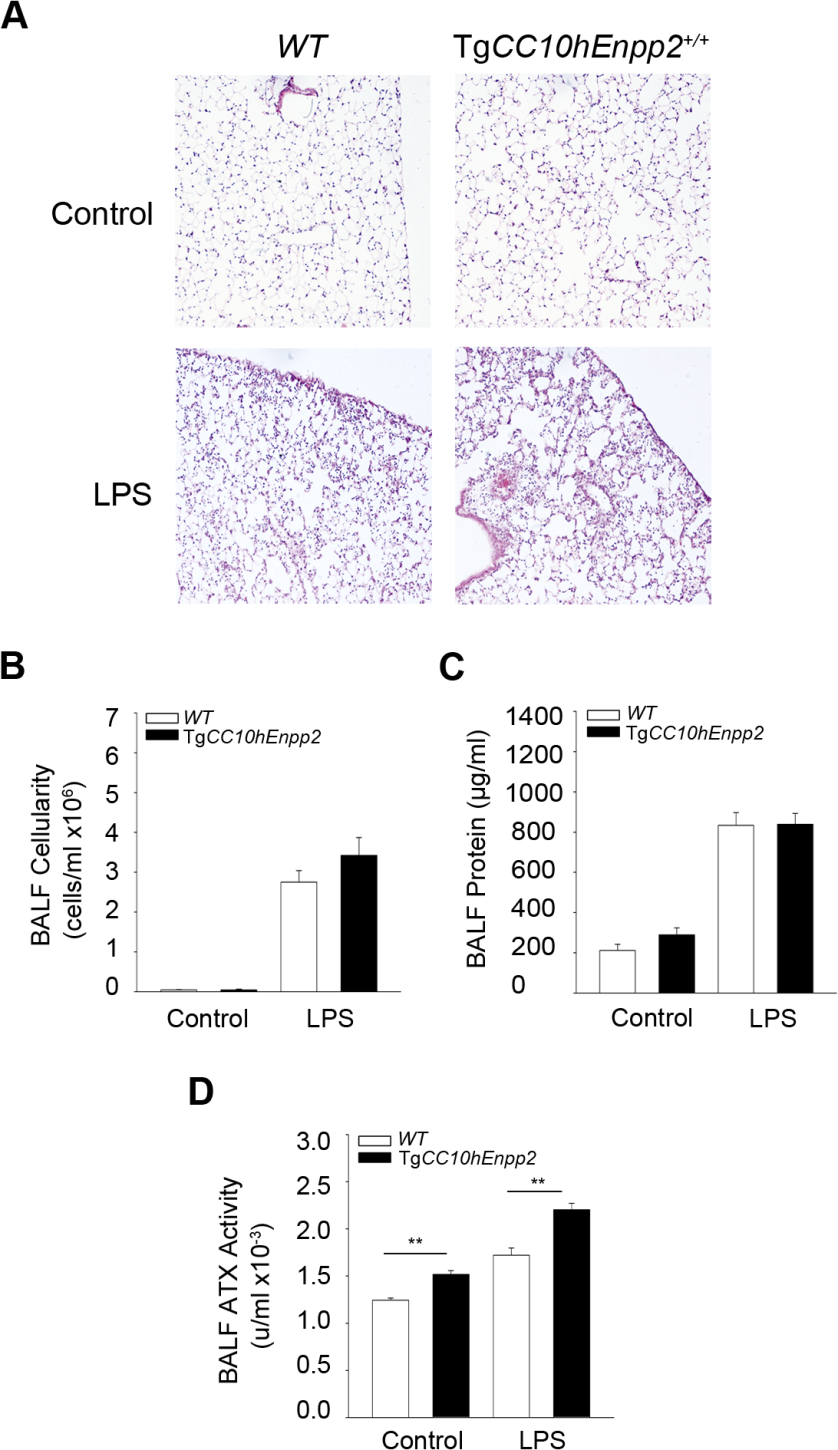
Genetic overexpression of ATX from the bronchial epithelium has minor effects in ALI development. Transgenic (Tg*CC10hEnpp2* ^*+/+*^) mice overexpressing hATX in the bronchial epithelium and littermate wild type mice were administered aerosolized LPS to induce ALI and were sacrificed 24 hours later (n = 3–10; a representative experiment out of two is shown). A-C. Histological analysis (A) and BALF measurements (B & C) indicated no significant changes in the lungs of mice overexpressing ATX expression in the bronchial epithelium in comparison to their wild type littermates. D. Increased enzyme activity of ATX in the transgenic mice.

To examine whether bronchial epithelial cell-derived hATX has any effect in LPS-induced ALI, LPS was administered in homozygous Tg*CC10hEnpp2* ^*+/+*^ mice (L16), which are healthy and fertile and exhibit no overt lung phenotype at 8–10 weeks after birth (Fig 4A). The moderate overexpression of ATX from bronchial epithelial cells had minor effects in exacerbating disease symptoms such as tissue damage (Fig 4A), neutrophilic inflammation (Fig 4B) and pulmonary edema (Fig 4C), although an opposite trend in disease severity could be observed in comparison to mice with bronchial epithelial deletion of ATX (Fig 2).

Therefore, bronchial epithelial expression of ATX does not seem to have a major role in LPS-induced, acute inflammation and lung injury, as opposed to its role in BLM-induced chronic pulmonary inflammation and fibrosis [18], suggesting a differential involvement of ATX/LPA in acute vs chronic pulmonary inflammation.

### Macrophage-specific ATX expression does not contribute to ALI pathogenesis

**Fig 5:**
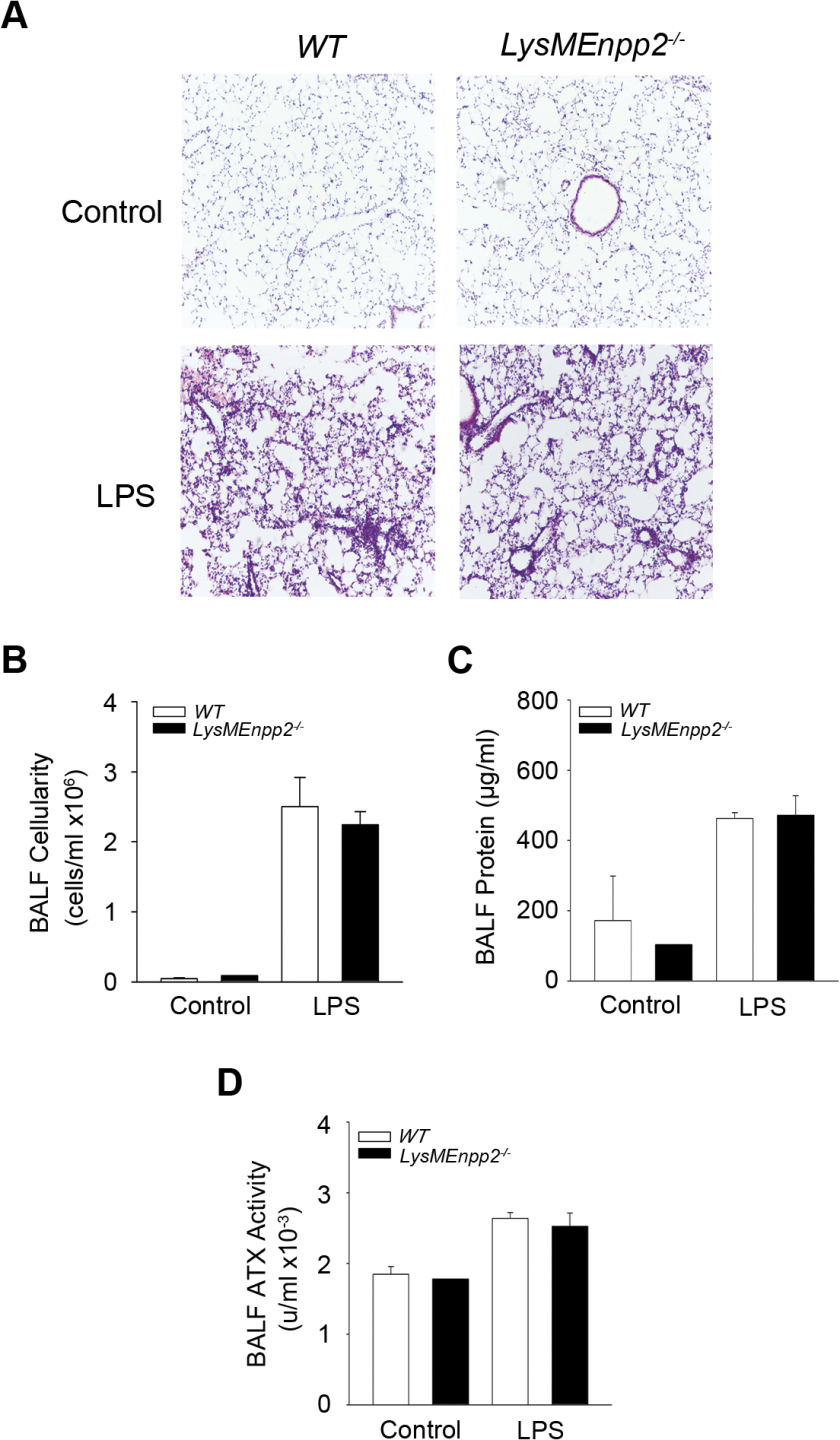
Deletion of ATX from myeloid cells had no effects in ALI development. Aerosolized LPS was administered in mice where ATX was deleted from the myeloid cells (LysMEnpp2^-/-^) and wild type littermate mice. Mice were sacrificed 24 hours later. A-C. Histological analysis (A) and BALF measurements (B & C) showed no differences in inflammation and edema between mice lacking ATX expression from myeloid cells and their wild type littermates. D. Conditional deletion of ATX from myeloid cells had no effects in BALF ATX activity.

To examine if myeloid cell derived ATX contributes to the pathogenesis of ALI, the conditional knock out mouse for ATX (*Enpp2* ^*n/n*^)[24] was crossed with a transgenic mouse strain (*LysM-Cre*) expressing the Cre recombinase under the control of the mouse Lysozyme M (*LysM*) promoter, which achieves a recombination efficiency close to 100% in granulocytes and 83–98% in macrophages [25]. No differences were observed in BALF ATX activity between *LysMEnpp2* ^-/-^ mice and their wild type littermates (Fig 5D), indicating no contribution of macrophages to BALF ATX levels upon LPS challenge. Accordingly, no differences were observed in all disease indices upon genetic deletion of ATX from macrophages (Fig 5), excluding a role of inflammatory macrophage derived ATX in LPS-induced acute lung inflammation in the lung, especially given the relatively few macrophages (<3%) in BALF infiltrates 24 hours post LPS [26, 27]. On the contrary macrophage derived ATX was shown to be important for the development of BLM-induced chronic pulmonary inflammation and fibrosis [18], where macrophages are the most abundant (>60%) infiltrating cell type during disease development [37].

### Increased systemic ATX levels exacerbate acute pulmonary inflammation

**Fig 6:**
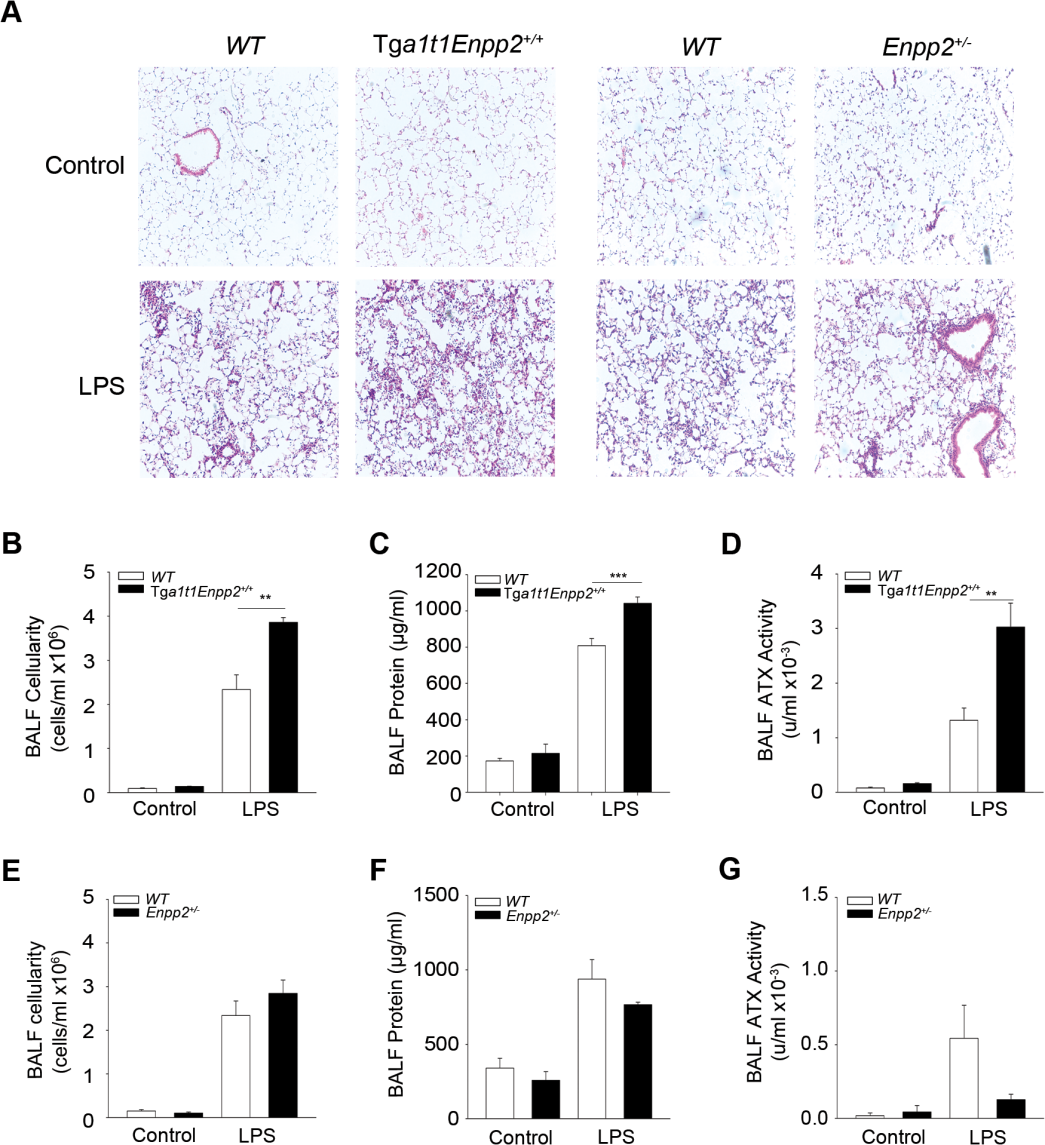
Systemic overexpression of ATX exacerbates ALI. Transgenic (Tg*a1t1Enpp2* ^*+/+*^) or heterozygous complete knock-out (Enpp2^+/-^) mice and their corresponding wild type littermate mice were administered with aerosolized LPS to induce lung injury and were sacrificed 24 hours later (n = 2–6; a representative experiment out of two is shown). A-D. Histological analysis (A) and BALF measurements (B & C) showed increased inflammation and edema in the lungs of the transgenic mice with systemic overexpression of ATX as a result of increased BALF ATX activity (D). On the contrary, histological analysis (A) and BALF measurements (E-G) showed non-significant effects in the lungs of the heterozygous knock-out mice as a result of decreased ATX activity levels.

Genetic deletion of ATX from bronchial epithelial cells, although diminished, did not attenuate pulmonary BALF ATX levels suggesting that a major part of BALF ATX is derived from the circulation, through the possible relaxation of endothelial and epithelial barriers upon LPS-induced ALI [4]. Therefore, and to investigate a possible role of circulating ATX in LPS/ALI, we next examined whether systemic fluctuations of ATX could modulate ALI pathogenesis. LPS was administered to homozygous transgenic mice overexpressing ATX in the liver driven by the human α1- antitrypsin inhibitor (*a1t1*) promoter (Tg*a1t1Enpp2*; up to 200% of normal plasma ATX/LPA levels) [38], as well as to the heterozygous complete knock out mouse for ATX (*Enpp2* ^*+/-*^; 50% of normal plasma ATX/LPA levels)[24]. Chronically elevated serum ATX levels in Tg*a1t1Enpp2* mice increased LPS-induced tissue damage (Fig 6A), BALF neutrophilic infiltration (Fig 6B) and pulmonary edema (Fig 6C) in comparison to their wild type littermates. However, reduced serum ATX levels in *Enpp2* ^*+/-*^ mice had only minor effects in LPS-induced ALI (Fig 6A, 6E and 6F), suggesting that a 50% reduction of systemic normal levels of ATX is not sufficient to confer resistance to ALI. Therefore, systemic, chronic exposure to increased ATX/LPA levels seems to predispose to and/or promote acute inflammation and ALI.

Noteworthy, systemic ATX levels were shown not to play a role in the development of modelled chronic inflammatory diseases, such as pulmonary fibrosis and rheumatoid arthritis where local ATX expression was shown to be the crucial event [18, 28], further suggesting a differential role of ATX/LPA in acute versus chronic inflammation.

### Pharmacologic inhibition of ATX does not alleviate ALI

**Fig 7:**
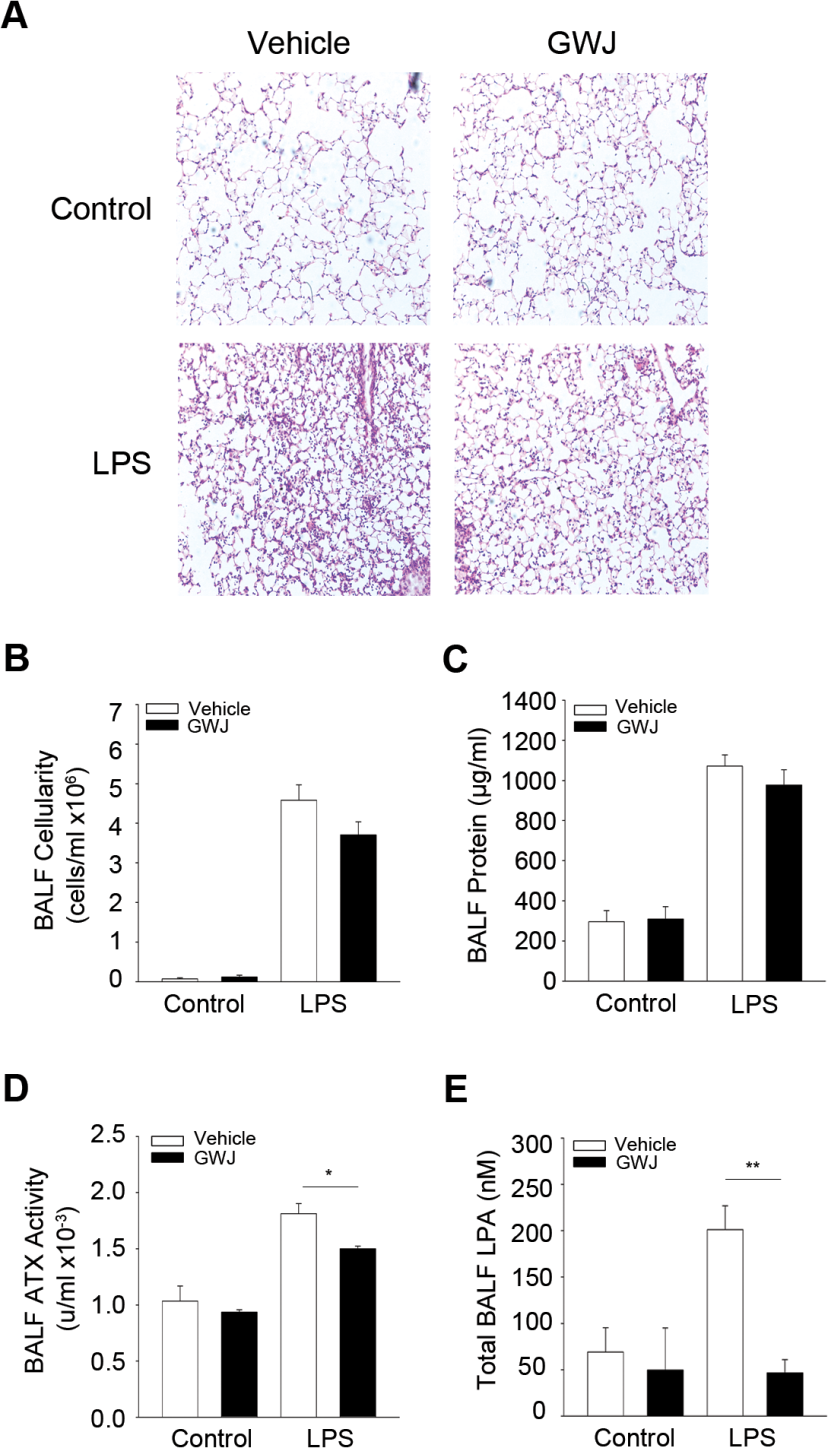
Pharmacologic inhibition of ATX has no effects in ALI development. GWJ-A-23 was injected intraperitoneally before challenging mice with aerosolized LPS. Mice were sacrificed 24 hours later (n = 3–7; a representative experiment out of two is shown). A-C. Histological analysis (A) and indicated BALF measurements (B,C) suggested minor effects in ALI development, despite decreased BALF ATX activity (D) and the corresponding BALF LPA levels (E).

It has been previously reported that pharmacologic inhibition of ATX using GWJ-A-23, a nanomolar ATX inhibitor [39, 40], attenuated both the development of BLM-induced pulmonary fibrosis [18], as well as triple-allergen (DRA)-induced asthma in mice [40]. Therefore, we next investigated the therapeutic potential of ATX inhibition in ALI. GWJ-A-23 was injected intraperitoneally just before LPS administration and disease indices were examined in comparison with vehicle treated littermate mice. As shown in Fig 7, ATX inhibition (Fig 7D) and reduction of LPA levels (Fig 7E) did not significantly attenuate ALI severity, as reflected in all disease indices (Fig 7A–7C), in agreement with the studies in *Enpp2* ^*+/-*^ mice. Likewise, pharmacologic antagonism of LPAR1 and 3 with ki16425 had minor effects in inflammation (<25%) and no effect in pulmonary edema [35]. Therefore, the results further confirm a differential role of ATX in acute versus chronic inflammation and suggest no therapeutic potential of targeting the ATX/LPA axis in ALI/ARDS.

## Conclusions

LPS administration to wt C57Bl6 mice resulted in increased ATX/LPA levels in BALFs, as previously reported [33, 35]. However, genetic deletion of ATX from bronchial epithelial cells or pharmacologic ATX inhibition, had minor effects in ALI pathology, as opposed to BLM-induced chronic pulmonary inflammation and fibrosis [18], suggesting a differential involvement of ATX/LPA in acute and chronic inflammation.

Similarly, the genetic deletion or pharmacologic antagonism of LPAR1 had no effect in vascular leak and edema upon LPS administration [35], the major hallmark of LPS/ALI-ARDS (and minimal, <25%, effects in inflammation, possibly due to genetic background differences of control mice). On the contrary, LPA/LPAR1-induced vascular leak was the main attribute (together with fibroblast recruitment) of the observed protection from BLM-induced chronic pulmonary inflammation and fibrosis upon LPAR1 genetic deletion [20](where no inflammatory changes were observed, especially in early time points) or pharmacologic inhibition [19], further supporting a differential role of ATX/LPA in acute vs chronic inflammation. The differences in LPA/LPAR1-mediated endothelial barrier functions in acute and chronic pulmonary inflammatory animal models suggest that the reported effects of LPA in endothelial permeability may need chronic exposure of target cells. Indeed, LPA effects in pulmonary endothelial permeability were found to increase with time (and of course concentration)[41]. Accordingly, chronically elevated serum ATX levels in Tg*a1t1Enpp2* mice increased LPS-induced acute lung injury by increasing both vascular leak and inflammation (Fig 6). On the contrary the systemic levels of ATX/LPA had no effect in chronic pulmonary inflammation and edema [18], perhaps due to the local expression of ATX leading to chronic LPA exposure of endothelial cells and a terminal increase of endothelial permeability that cannot be modulated further.

The likely differential involvement of ATX/LPA in acute inflammation could possibly be also attributed in part to macrophage specific ATX expression. Very few (<3%) macrophages infiltrate LPS challenged lungs and our results have shown that they don’t contribute to the BALF ATX load nor to disease development (Fig 5). However, reducing macrophage (the most abundant, >60%, infiltrating cell type) ATX expression in BLM-induced chronic pulmonary inflammation and fibrosis reduced both ATX BALF load and disease development [18].

Moreover, a more prominent role of ATX/LPA in chronic inflammation is consistent with their role in cancer [16], given the increasing links of chronic inflammation and carcinogenesis. Conditional deletion of ATX from bronchial epithelial cells that although had minor effects in LPS-induced ALI, attenuated the development of both pulmonary inflammation and fibrosis [18], as well urethane-induced lung cancer [42]. The issue is currently investigated in a more suitable context for such studies, namely liver pathogenesis.

Finally, any biological outcome of increased ATX/LPA levels would depend on the abundance and activity of the different LPA receptors in different cell types participating in the different phases of an inflammatory response, especially given the reported anti-inflammatory effects of LPA/LPAR2 on innate immune responses in the lung [43] and the suggested roles of LPA in the regulation of adaptive immune responses [44]. Therefore, the complete understanding of the involvement of ATX/LPA in the various forms of inflammation will require precise knowledge of the spatiotemporal regulation of ATX and LPA receptors expression, as well as the cell-specific LPA effects in the different cell types involved in inflammatory responses.
